# Supplementary material for: Intermolecular charge transfer enhances the performance of molecular rectifiers
Source: Sci Adv. 2022 Aug 5;8(31):eabq7224. doi: 10.1126/sciadv.abq7224 (PMC9355360; doi:10.1126/sciadv.abq7224)
Supplement: Supplementary file 1 — Supplementary Text Figs. S1 to S23 Table S1 [file sciadv.abq7224_sm.pdf]

Science Advances
AAAS

Supplementary Materials for

Intermolecular charge transfer enhances the performance of molecular rectifiers

Ryan P. Sullivan *et al.*

Corresponding author: Oana D. Jurchescu, jurchescu@wfu.edu

Sci. Adv. **8**, eabq7224 (2022)
DOI: 10.1126/sciadv.abq7224

This PDF file includes:

Supplementary Text
Figs. S1 to S23
Table S1

Supplementary Text

Material Synthesis

methyl (E)-4-(((3-(triethoxysilyl)propyl)imino)methyl)benzoate (CMPTM)
Methyl 4-formyl benzoate (0.075g, 0.457 mmol) and 3-(triethoxysilyl)propan-1-amine (APTES) (0.103g, 0.461 mmol) were reacted as described in the general procedure to yield a viscous light-yellow liquid (0.137g, 0.373 mmol, 82%). ^{1}H NMR (400 MHz, $CDCl_3$) δ 8.25 (s, 1H), 8.00 (m, 2H), 7.72 (m, 2H), 3.86 (s, 3H), 3.76 (q, *J* = 7.0 Hz, 6H), 3.58 (td, *J* = 6.9, 1.3 Hz, 2H), 1.83 – 1.71 (m, 2H), 1.16 (t, *J* = 7.0 Hz, 9H), 0.66 – 0.57 (m, 2H). ^{13}C NMR (101 MHz, $CDCl_3$) δ 165.70, 159.06, 139.21, 130.63, 128.82, 126.89, 63.40, 57.38, 51.22, 23.17, 17.29, 7.05. HRMS (APCI-ion trap) m/z: $[M+H]^+$ Calc for $C_{18}H_{29}NO_5SiH$: 368.189327; Found: 368.18891.

methyl (E)-2-methyl-4-(((3-(triethoxysilyl)propyl)imino)methyl)benzoate (CMMPTM**)**
Methyl 4-formyl-2methyl benzoate (0.089g, 0.500mmol) and 3-(triethoxysilyl)propan-1-amine (APTES) (0.111g, 0.501mmol) were reacted as described in the general procedure to yield an off-white flaky solid (0.182g, 0.477mmol, 56 %) ^{1}H NMR (400 MHz, $CDCl_3$) δ 8.20 (d, *J* = 1.3 Hz, 1H), 7.87 (d, *J* = 8.0 Hz, 1H), 7.54 (d, *J* = 1.6 Hz, 1H), 7.49 (dd, *J* = 8.1, 1.7 Hz, 1H), 3.83 (s, 3H), 3.76 (q, *J* = 7.0 Hz, 6H), 3.56 (td, *J* = 6.9, 1.4 Hz, 2H), 2.56 (s, 3H), 1.83 – 1.72 (m, 2H), 1.16 (t, *J* = 7.0 Hz, 9H), 0.65 – 0.58 (m, 2H). ^{13}C NMR (101 MHz, $CDCl_3$) δ 167.69, 160.24, 140.58, 139.15, 131.13, 130.93, 130.92, 125.37, 64.46, 58.40, 51.93, 24.20, 21.65, 18.32, 8.07. HRMS (APCI-ion trap) m/z: $[M+H]^+$ Calc for $C_{19}H_{31}NO_5SiH$: 382.20498; Found: 382.20518

(E)-1-(4-(methylthio)phenyl)-N-(3-(triethoxysilyl)propyl)methanimine (MPTM)
4-(methylthio) benzaldehyde (0.153g, 1mmol) and 3-(triethoxysilyl)propan-1-amine (APTES) (0.223g, 1.01mmol) were combined with Na_2SO_4 (3.5g) in DCM (30 mL) and refluxed at 75-80 ^{O}C for 26 hours and then stirred for another 24 hours at room temperature. Workup was carried out as described in the general procedure, yielding a gold oil (0.288g, 0.623mmol, 62 %). ^{1}H NMR (400 MHz, $CDCl_3$) δ 8.13 (s, 1H), 7.57 (d, *J* = 8.0 Hz, 2H), 7.19 – 7.16 (m, 2H), 3.75 (q, *J* = 7.0 Hz, 6H), 3.52 (td, *J* = 6.8, 1.3 Hz, 2H), 2.44 (s, 3H), 1.82 – 1.70 (m, 2H), 1.15 (t, *J* = 7.0 Hz, 9H), 0.65 – 0.55 (m, 2H). ^{13}C NMR (101 MHz, $CDCl_3$) δ 160.47, 130.01, 128.46, 125.82, 125.21, 64.17, 58.38, 24.26, 18.32, 15.27, 8.02. HRMS (APCI-ion trap) m/z: $[M+H]^+$ Calc for $C_{17}H_{29}NO_3SSiH$: 356.17157; Found: 356.17101

(E)-1-(4-methoxyphenyl)-N-(3-(triethoxysilyl)propyl)methanimine (MTPTM)
4-methoxy benzaldehyde (0.137g, 1mmol) and 3-(triethoxysilyl)propan-1-amine (APTES) (0.223g, 1.01mmol) were combined with 3.5g Na_2SO_4 and refluxed at 75-80C for 26 hours to give a gold oil (0.230g, 0.676mmol, 68 %). ^{1}H NMR (400 MHz, $CDCl_3$) δ 8.12 (s, 1H), 7.64 – 7.54 (m, 2H), 6.88 – 6.83 (m, 2H), 3.77 (s, 3H), 3.753 (q, J=7Hz, 6H), 3.51 (td, *J* = 6.9, 1.3 Hz, 2H), 1.82 – 1.69 (m, 2H), 1.15 (t, *J* = 7.0 Hz, 9H), 0.66 – 0.55 (m, 2H) ^{13}C NMR (101 MHz, $CDCl_3$) δ 161.48, 160.35, 129.57, 129.32, 113.94, 64.26, 58.37, 55.34, 24.34, 18.32, 8.02. HRMS (APCI-ion trap) m/z: $[M+H]^+$ Calc for C17H29NO4SiH: 340.19441; Found:340.19356

(E)-1-phenyl-N-(3-(triethoxysilyl)propyl)methanimine (PTM)
benzaldehyde (0.107g, 1mmol) and 3-(triethoxysilyl)propan-1-amine (APTES) (0.222g, 1mmol) were reacted as described in the general procedure to give a clear oil (0.200g, 0.644mmol, 64%) ^{1}H NMR (400 MHz, $CDCl_3$) δ 8.20 (t, *J* = 1.3 Hz, 1H), 7.69 – 7.62 (m, 2H), 7.37 – 7.30 (m, 3H),

3.75 (q, J = 7.0 Hz, 6H), 3.55 (td, J = 7.0, 1.4 Hz, 2H), 1.82 – 1.70 (m, 2H), 1.15 (t, J = 7.0 Hz, 9H), 0.67 – 0.56 (m, 2H) ^{13}C NMR (101 MHz, $CDCl_3$) δ 161.05, 136.34, 130.47, 128.57, 128.05, 64.33, 58.38, 24.26, 18.32, 8.04. HRMS (APCI-ion trap) m/z: $[M+H]^+$ Calc fo $C_{16}H_{27}NO_3SiH$: 310.18384; Found: 310.18435

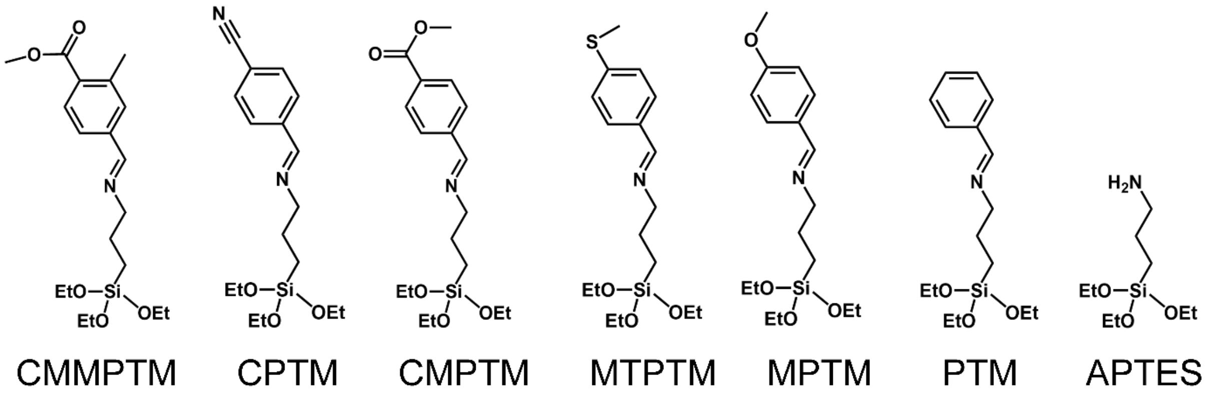

Fig. S1: Chemical structures of all rectifying molecules and the APTES additive. (E)-1-(4-carbomethoxy-3-methyl-phenyl)-N-(3-(triethoxysilyl)propyl)methanimine (CMMPTM), (E)-1-(4-cyanophenyl)-N-(3-(triethoxysilyl) propyl)methanimine (CPTM), (E)-1-(4-carbomethoxy-phenyl)-N-(3-(triethoxysilyl)propyl)methanimine (CMPTM), (E)-1-(4-(methylthio)phenyl)-N-(3-(triethoxysilyl)propyl)methanimine (MTPTM), (E)-1-(4-methoxyphenyl)-N-(3-(triethoxysilyl)propyl)methanimine (MPTM), (E)-1-phenyl-N-(3-(triethoxysilyl)propyl)methanimine (PTM), (3-Aminopropyl)triethoxysilane (APTES).

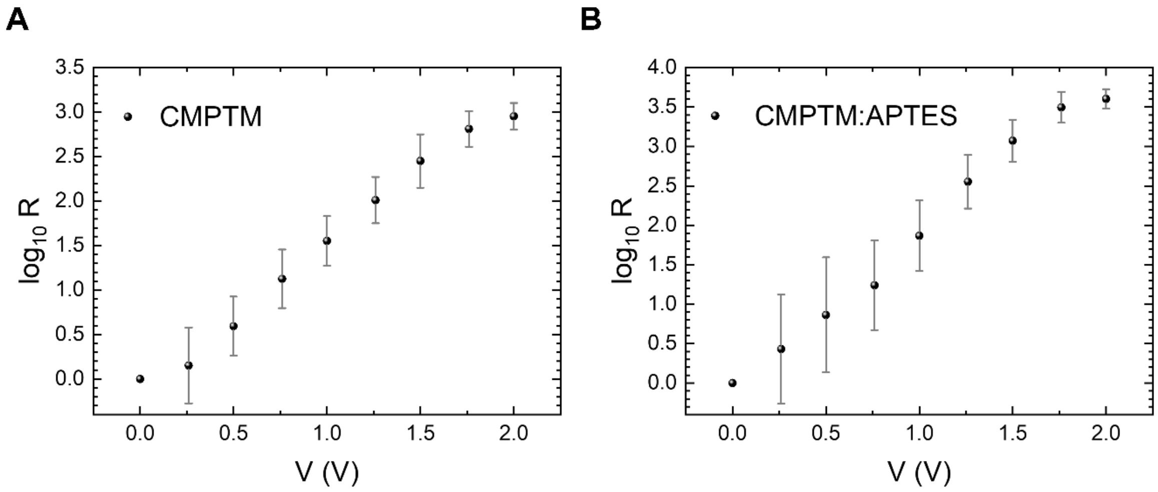

Fig. S2: Rectification ratio R vs applied voltage. (**A**) Molecular rectifiers based on SAMs of CMPTM. (**B**) Molecular rectifiers based on mixed SAMs of CMPTM:APTES.

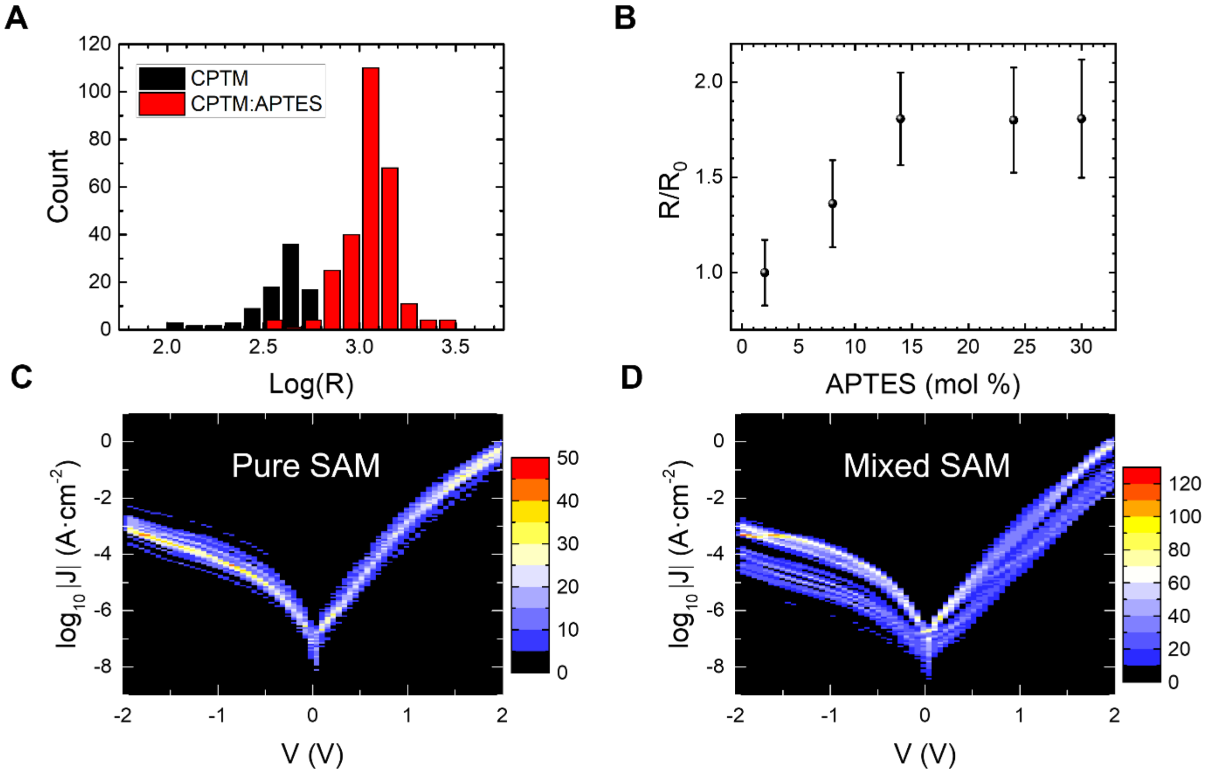

Fig. S3: Rectification properties of molecular diodes fabricated on CPTM and CPTM:APTES SAMs. (**A**) Histogram of rectification ratios for CPTM (black) and CPTM:APTES (red) SAMs. (**B**) Dependence of the rectification ratio R on the APTES concentration; the value is normalized by the rectification ratio in pure CPTM, R_0. (**C**) Current density versus voltage in pure CPTM rectifiers. (**D**) Current density versus voltage in CPTM:APTES rectifiers.

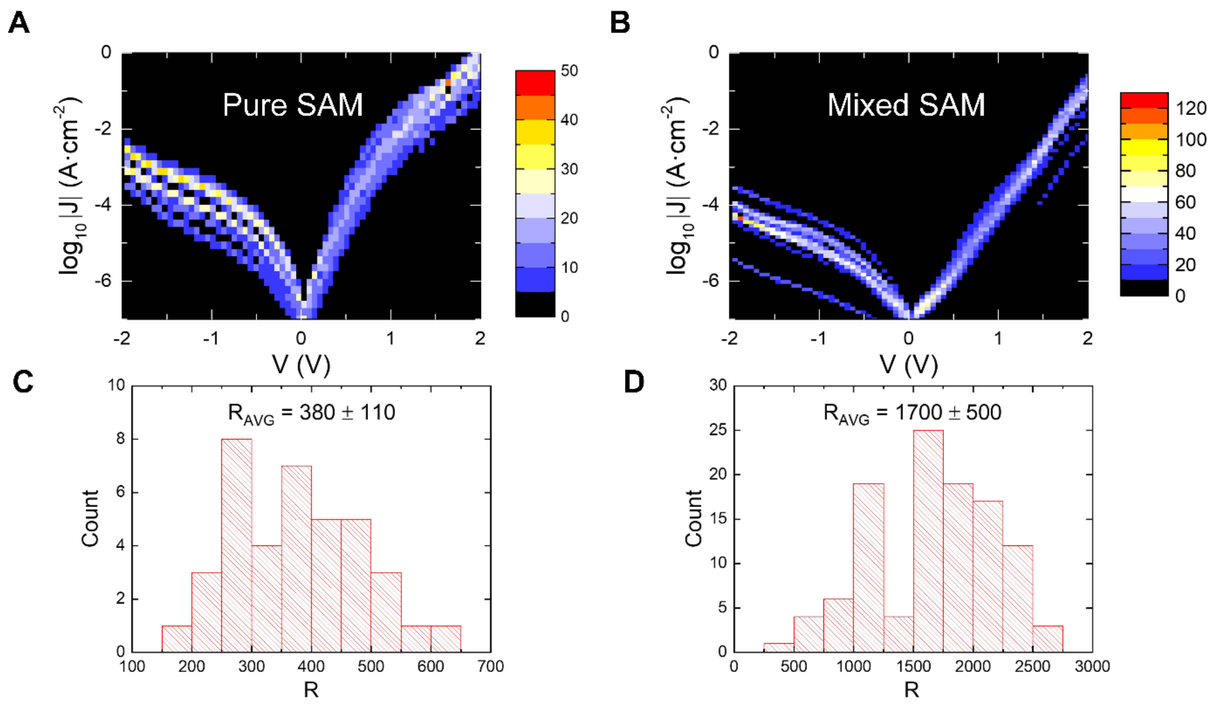

Fig. S4: Rectification properties of molecular diodes fabricated on CMMPTM and CMMPTM:APTES SAMs. (**A**) Current density versus voltage in pure CMMPTM rectifiers. (**B**) Current density versus voltage in CMMPTM:APTES rectifiers. (**C**) Histogram of rectification ratios in CMMPTM devices. The average rectification value is included in the inset. (**D**) Histogram of rectification ratios in CMMPTM:APTES devices. The average rectification value is included in the inset.

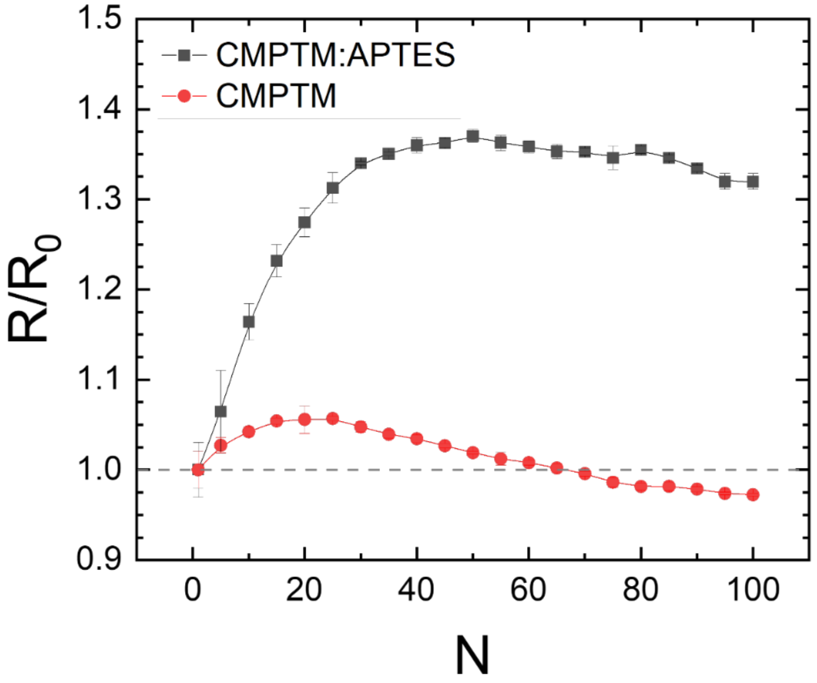

Fig. S5: Bias stress measurements in pure and mixed SAMs molecular rectifiers. The rectification ratio of devices fabricated on SAMs of CMPTM:APTES at 70:30 (black) and pure CMPTM (red) for 100 repeated measurements between +/- 2V.

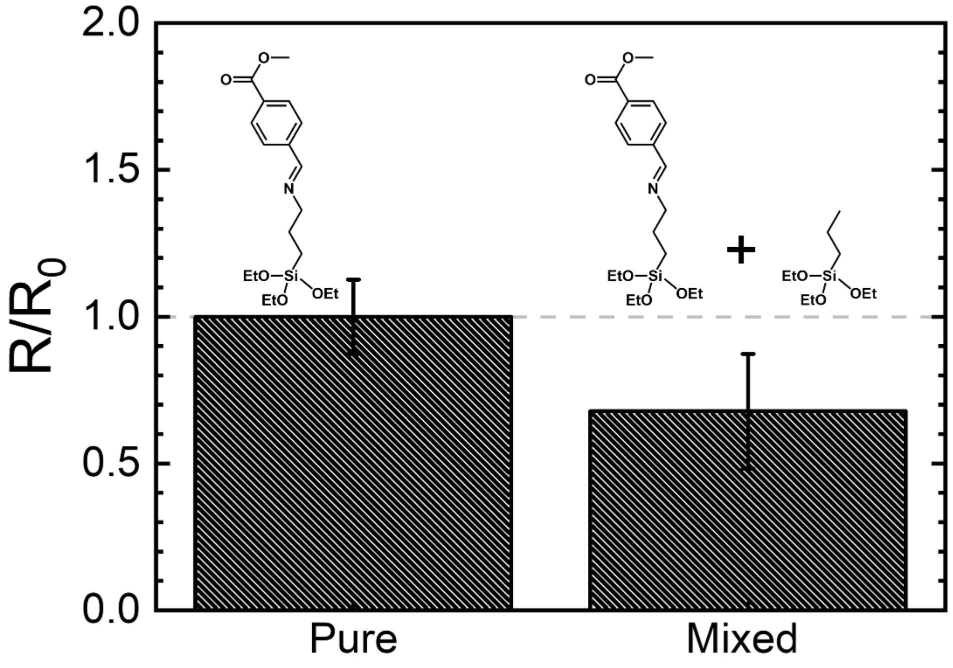

Fig. S6: Rectification ratio in CMPTM diluted with a SAM that does not promote charge transfer. Average rectification ratio normalized with respect to the value obtained in pure SAM in CMPTM and CMPTM mixed with the nonrectifying additive triethoxy(propyl)silane.

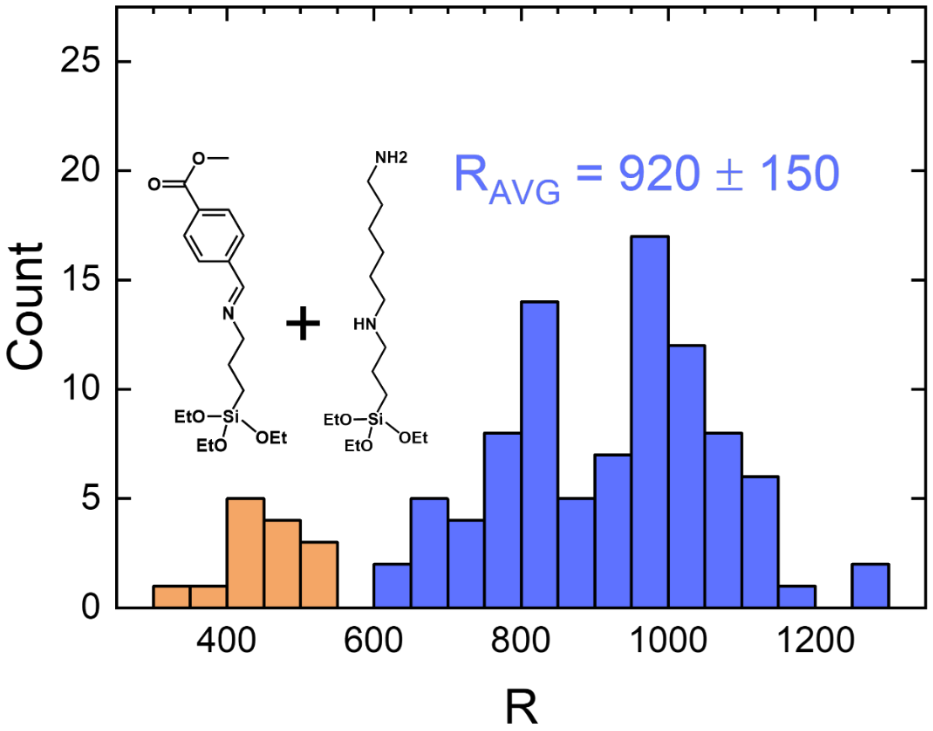

Fig. S7: Rectification properties of CMPTM mixed with N-(6-aminohexyl) aminomethyltriethoxysilane at a molar ratio of 70:30. Phase segregation is suggested by the presence of two distinct histograms illustrated in orange and blue. The average R listed in the inset corresponds to the blue data.

Molecule	σ_p	σ_{p-}	σ_{p+}
CPTM	0.66	1.0	0.66
CMPTM	-	0.75	0.49
CMMPTM	-	0.44	0.32
PTM	0.0	0.0	0.0
NH_2 (APTES)	-0.66	-0.15	-1.3
MPTM	-	-0.26	-0.78
MTPTM	-	0.06	-0.6

Table S1: Hammett constants for substituents on the terminating benzene rings for the specified molecules (*40*).

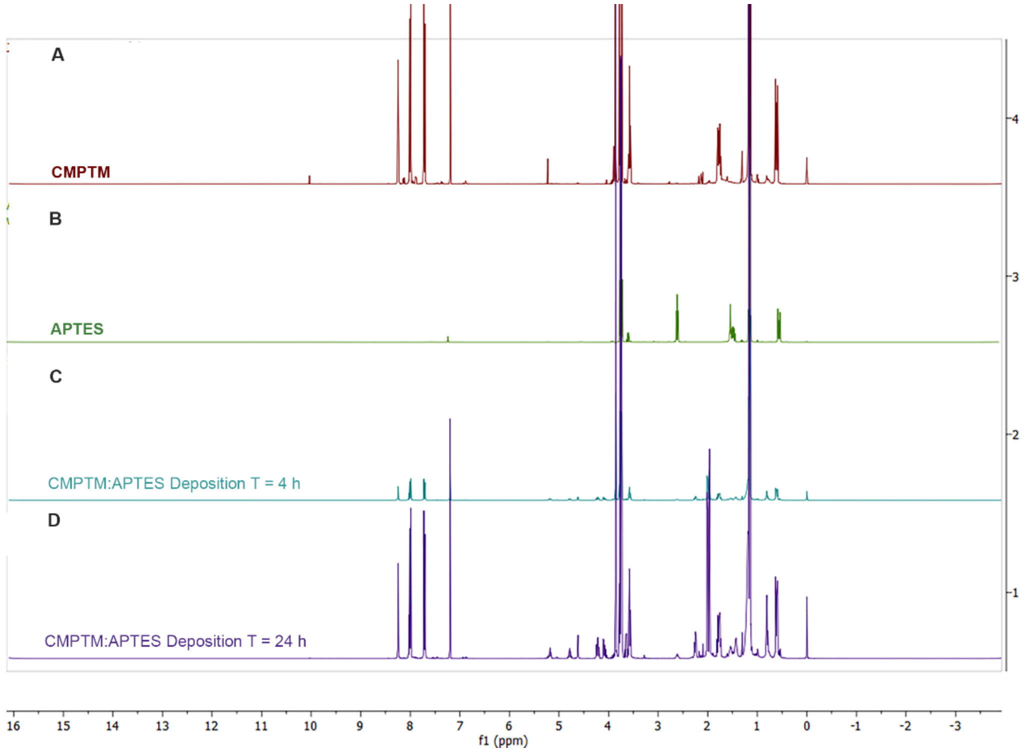

Fig. S8: HNMR spectra for CMPTM and APTES molecules. (**A**) CMPTM. (**B**) APTES. (**C**) CMPTM:APTES SAMs deposited on a silicon shard, the measurement was taken 4 hours after fabrication. (**D**) CMPTM:APTES SAMs deposited on a silicon shard, the measurement was taken 24 hours after fabrication.

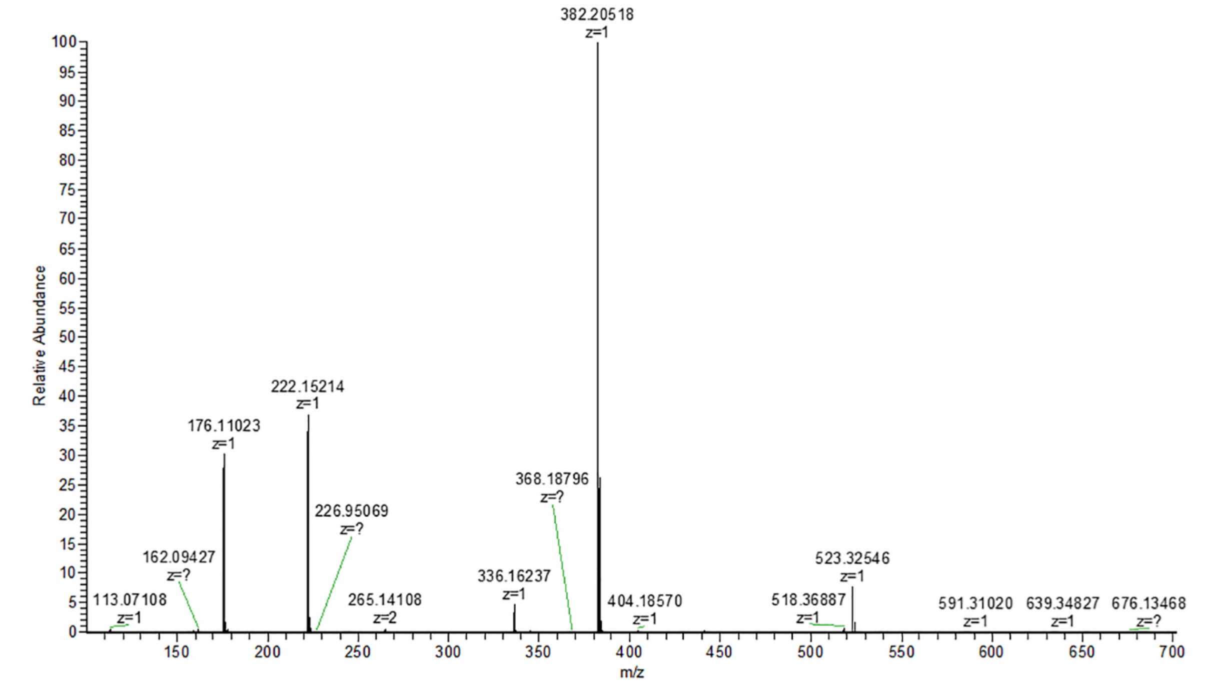

Fig. S9: HRMS spectrum of CMMPTM. HRMS spectrum of methyl (E)-2-methyl-4-(((3-(triethoxysilyl)propyl)imino)methyl)benzoate.

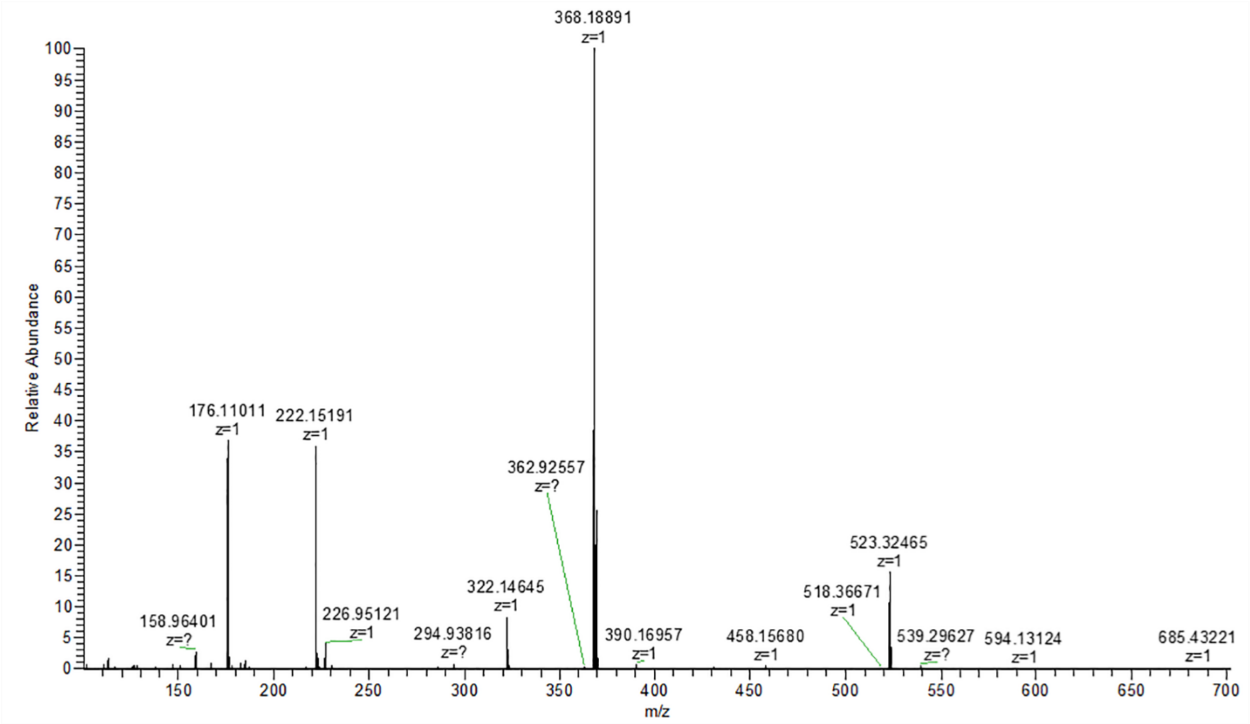

Fig. S10: HRMS spectrum of CMPTM. HRMS spectrum of methyl (E)-4-(((3-(triethoxysilyl)propyl)imino)methyl)benzoate.

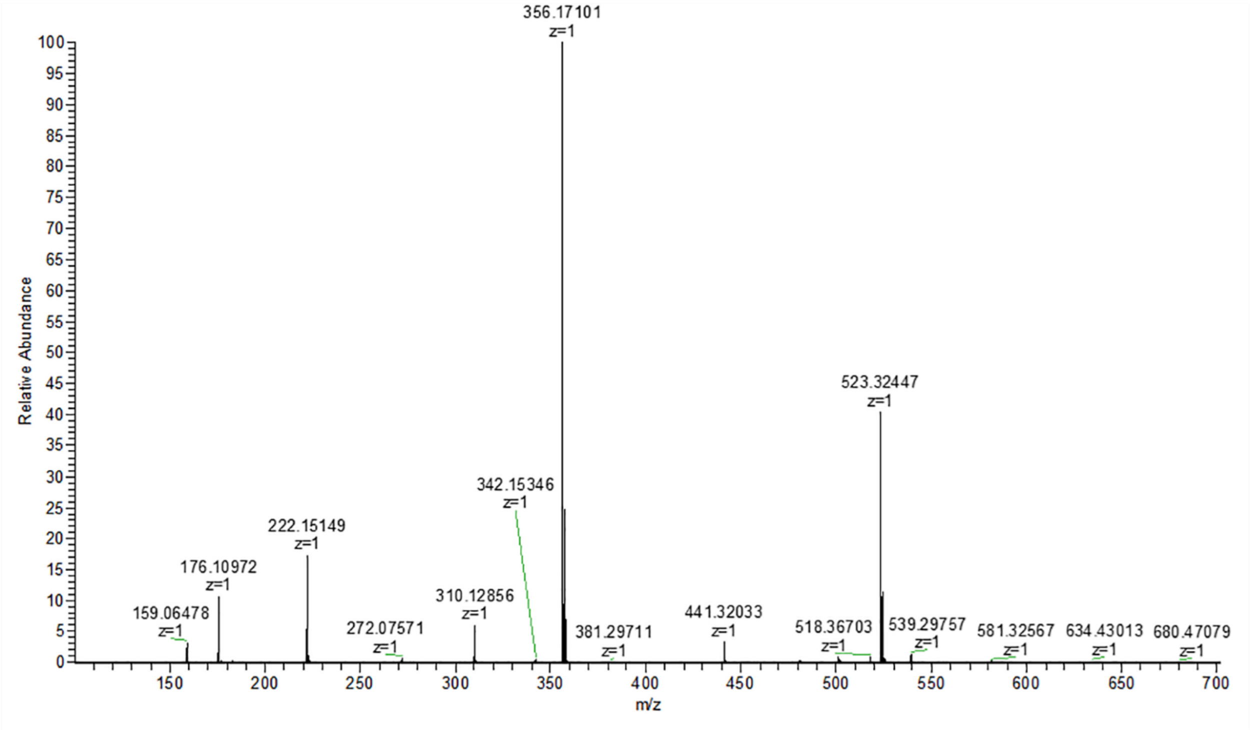

Fig. S11: HRMS spectrum of MPTM. HRMS spectrum of (E)-1-(4-(methylthio)phenyl)-N-(3-(triethoxysilyl)propyl)methanimine.

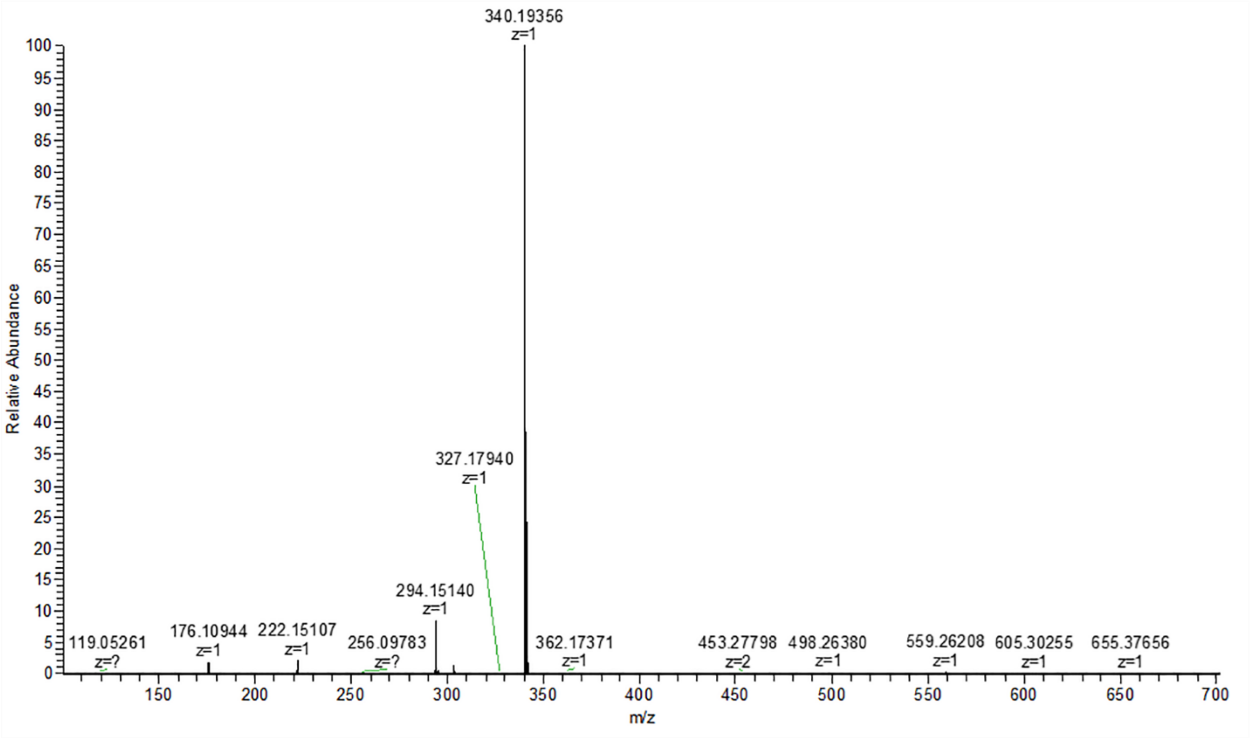

Fig. S12: HRMS spectrum of MTPTM. HRMS spectrum of (E)-1-(4-methoxyphenyl)-N-(3-(triethoxysilyl)propyl)methanimine.

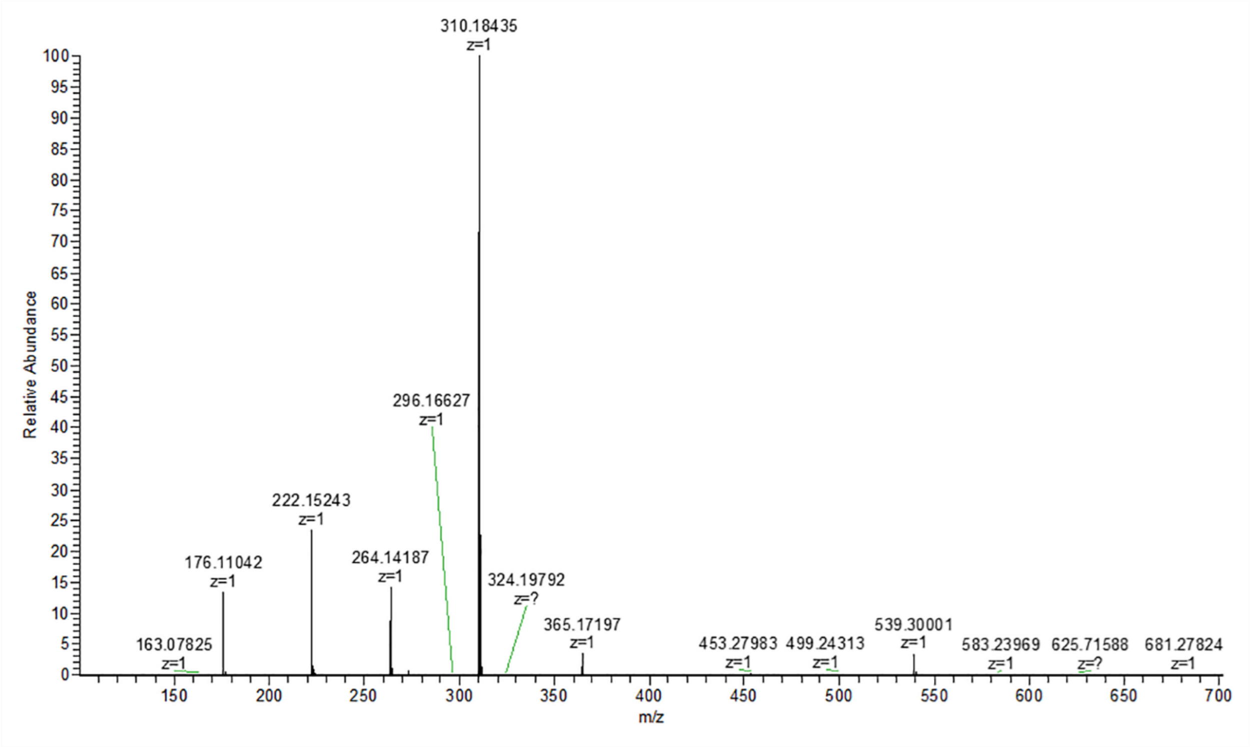

Fig. S13: HRMS spectrum of PTM. HRMS spectrum of (E)-1-phenyl-N-(3-(triethoxysilyl)propyl)methanimine.

.

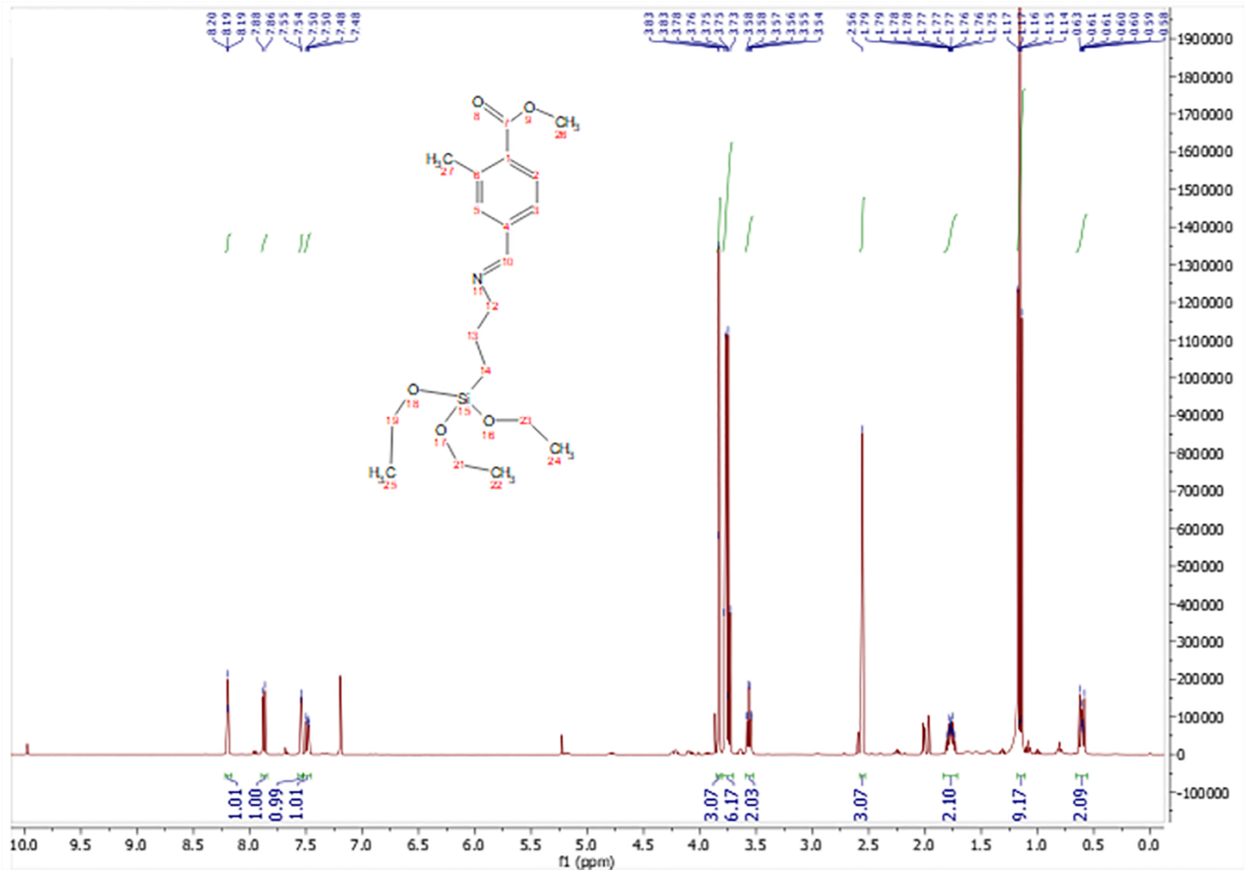

Fig. S14: ^{1}HNMR spectrum of CMMPTM. ^{1}HNMR spectrum of (E)-1-(4-carbomethoxy-3-methyl-phenyl)-N-(3-(triethoxysilyl)propyl)methanimine.

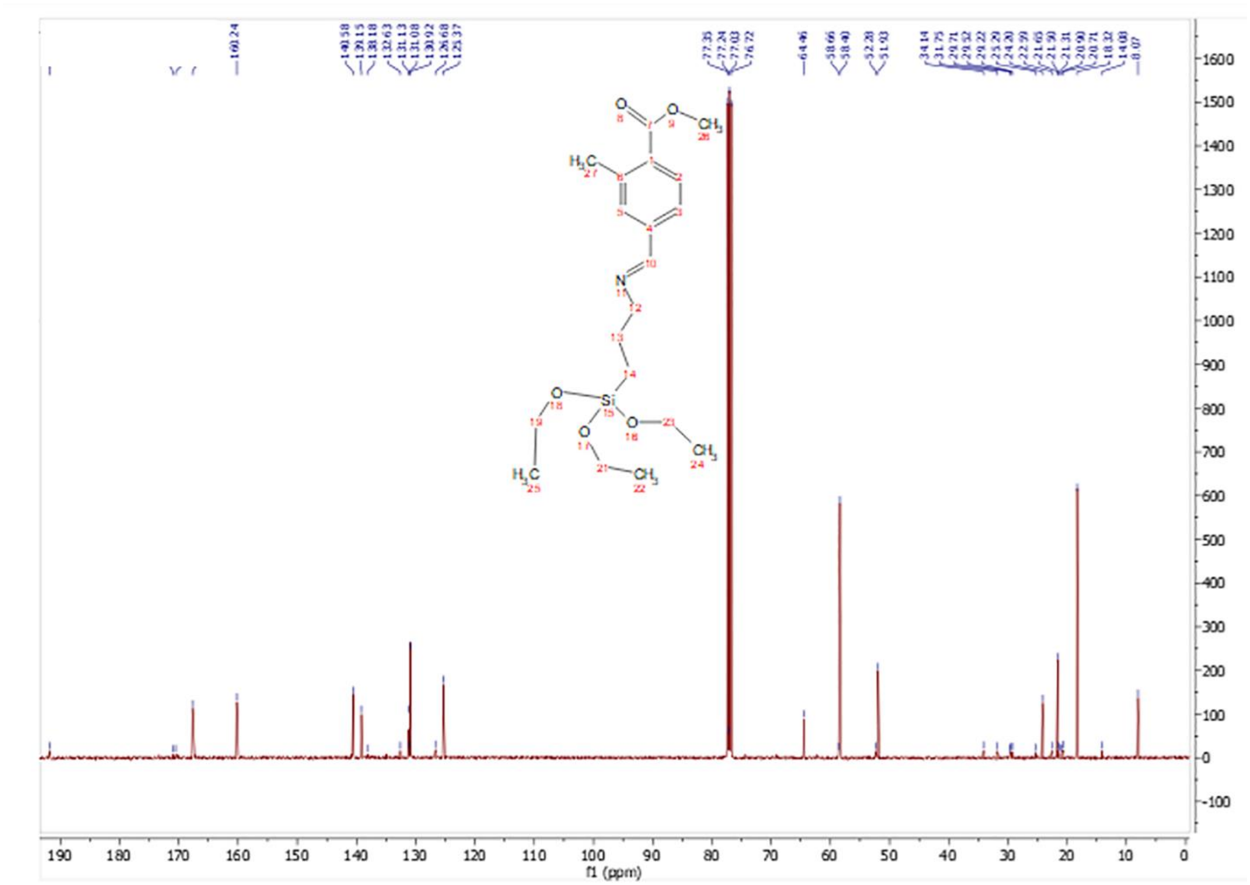

Fig. S15: ^{13}CNMR spectrum of CMMPTM. ^{13}CNMR spectrum of (E)-1-(4-carbomethoxy-3-methyl-phenyl)-N-(3-(triethoxysilyl)propyl)methanimine.

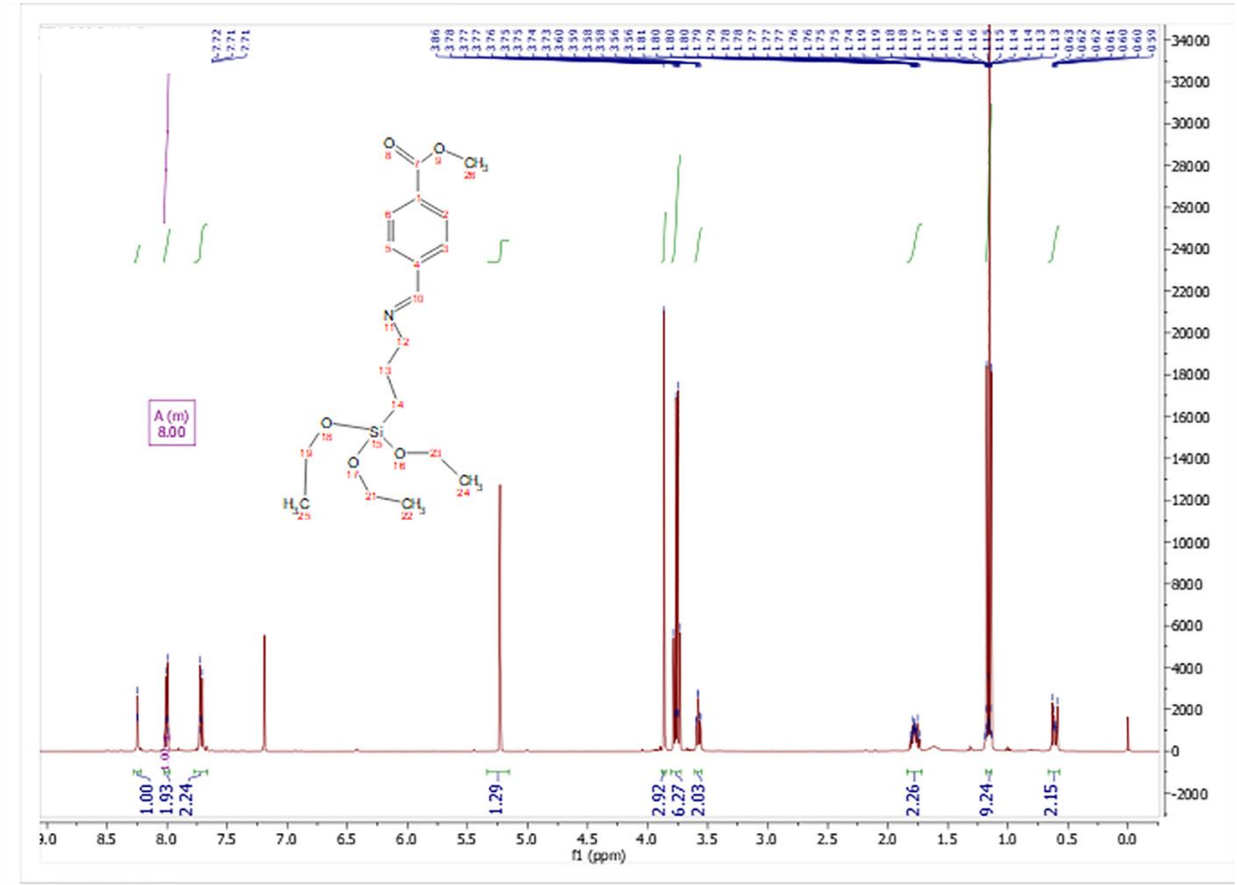

Fig. S16: ^{1}HNMR spectrum of CMPTM. ^{1}HNMR spectrum of (E)-1-(4-carbomethoxy-phenyl)-N-(3-(triethoxysilyl)propyl)methanimine.

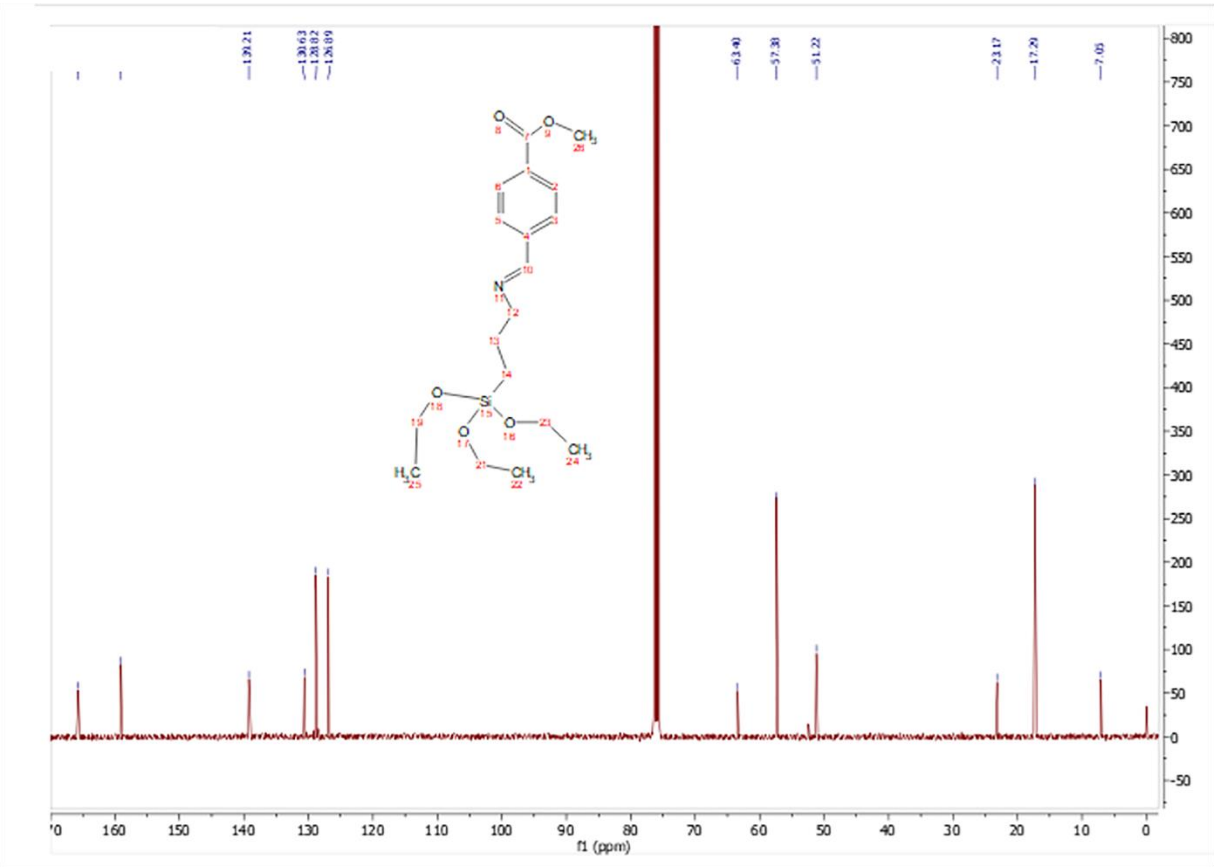

Fig. S17: ^{13}CNMR spectrum of CMPTM. ^{13}CNMR spectrum of (E)-1-(4-carbomethoxy-phenyl)-N-(3-(triethoxysilyl)propyl)methanimine.

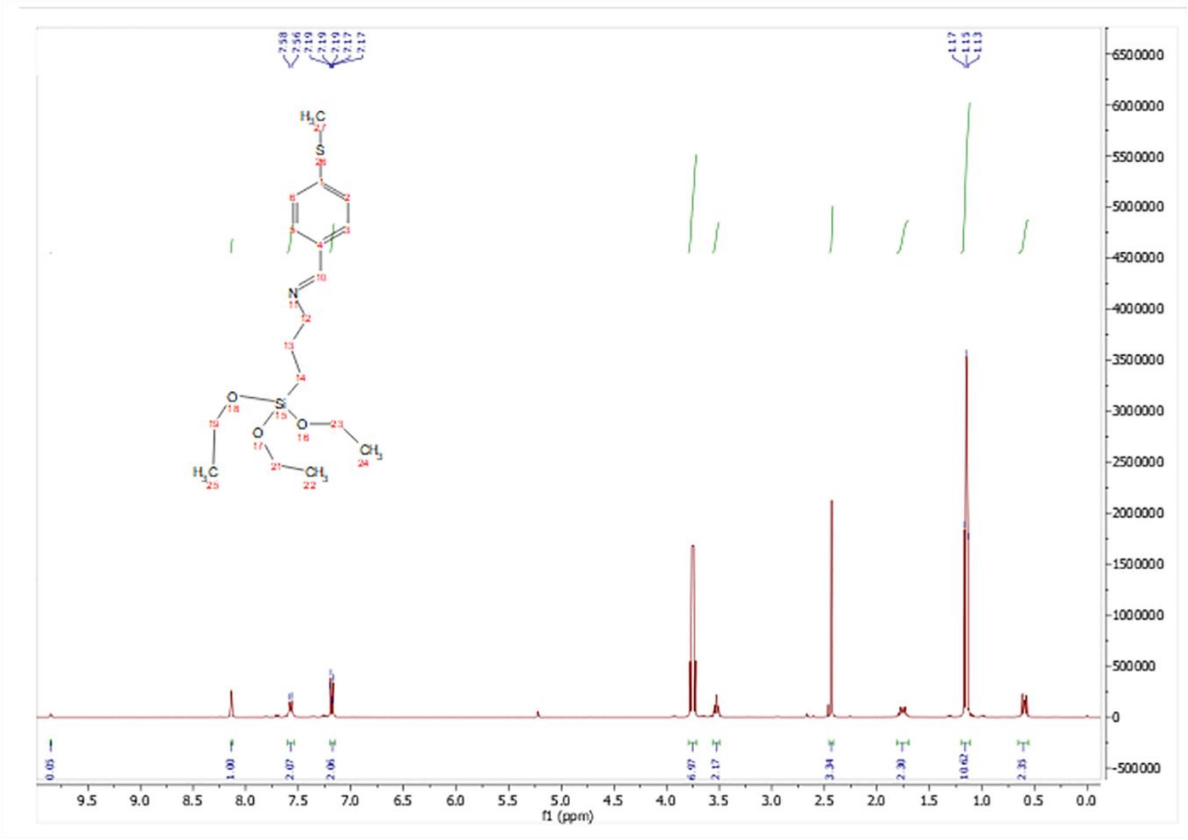

Fig. S18: ^{1}HNMR spectrum of MPTM. ^{1}HNMR spectrum of. (E)-1-(4-(methylthio)phenyl)-N-(3-(triethoxysilyl)propyl)methanimine.

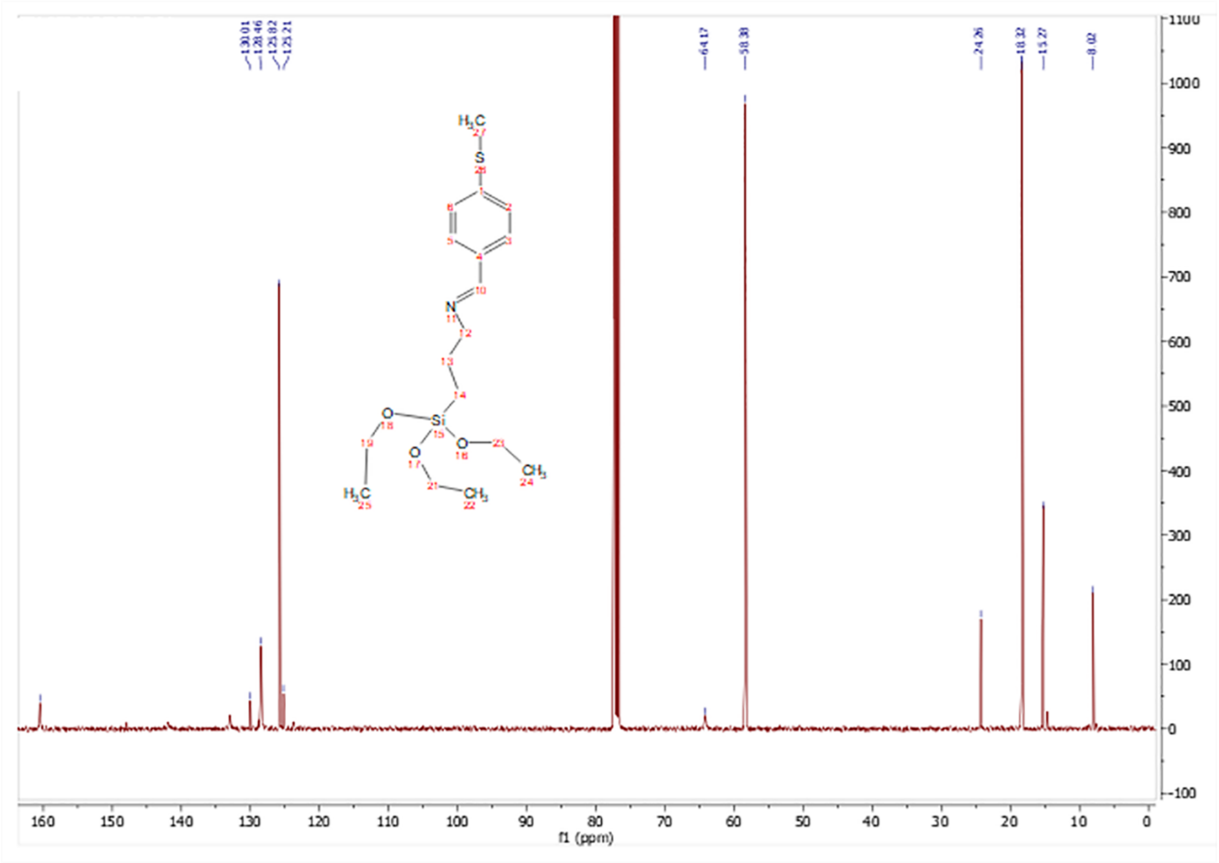

Fig. S19: ^{13}CNMR spectrum of MPTM. ^{13}CNMR spectrum of (E)-1-(4-(methylthio)phenyl)-N-(3-(triethoxysilyl)propyl)methanimine.

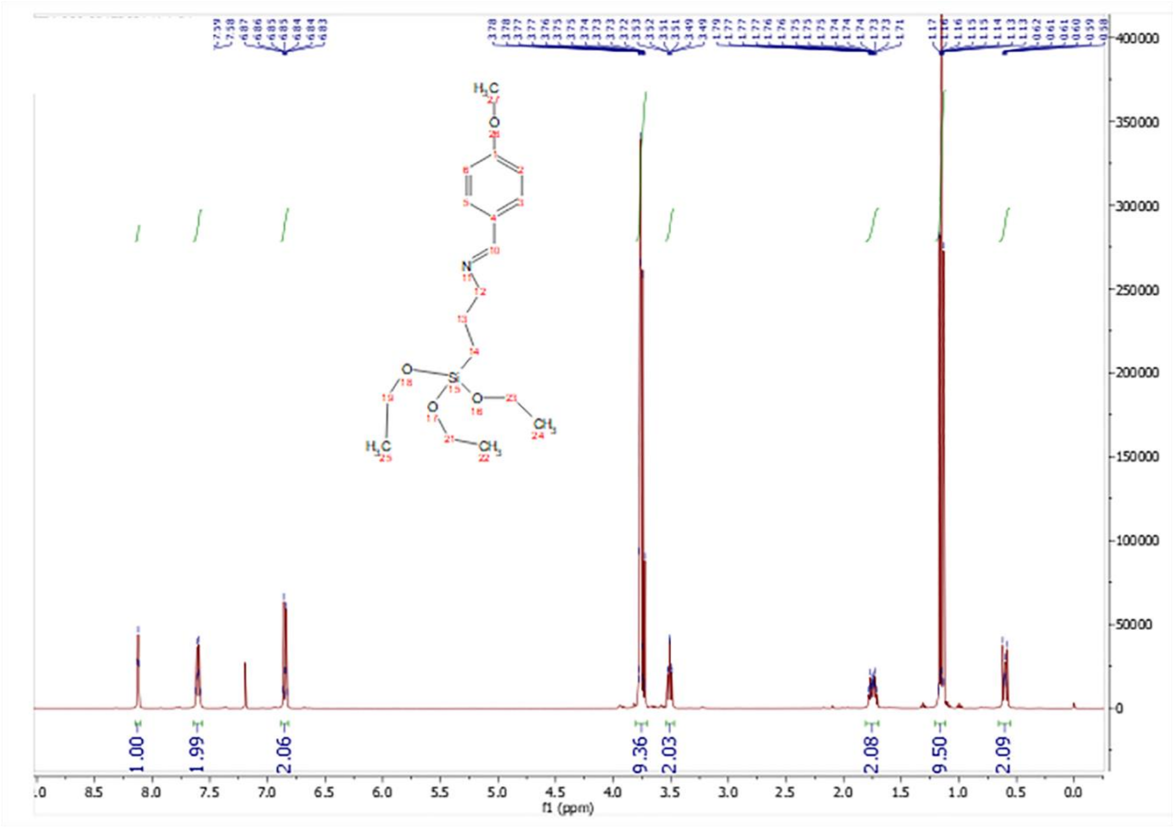

Fig. S20: ^{1}HNMR spectrum of MTPTM. ^{1}HNMR spectrum of (E)-1-(4-methoxyphenyl)-N-(3-(triethoxysilyl)propyl)methanimine.

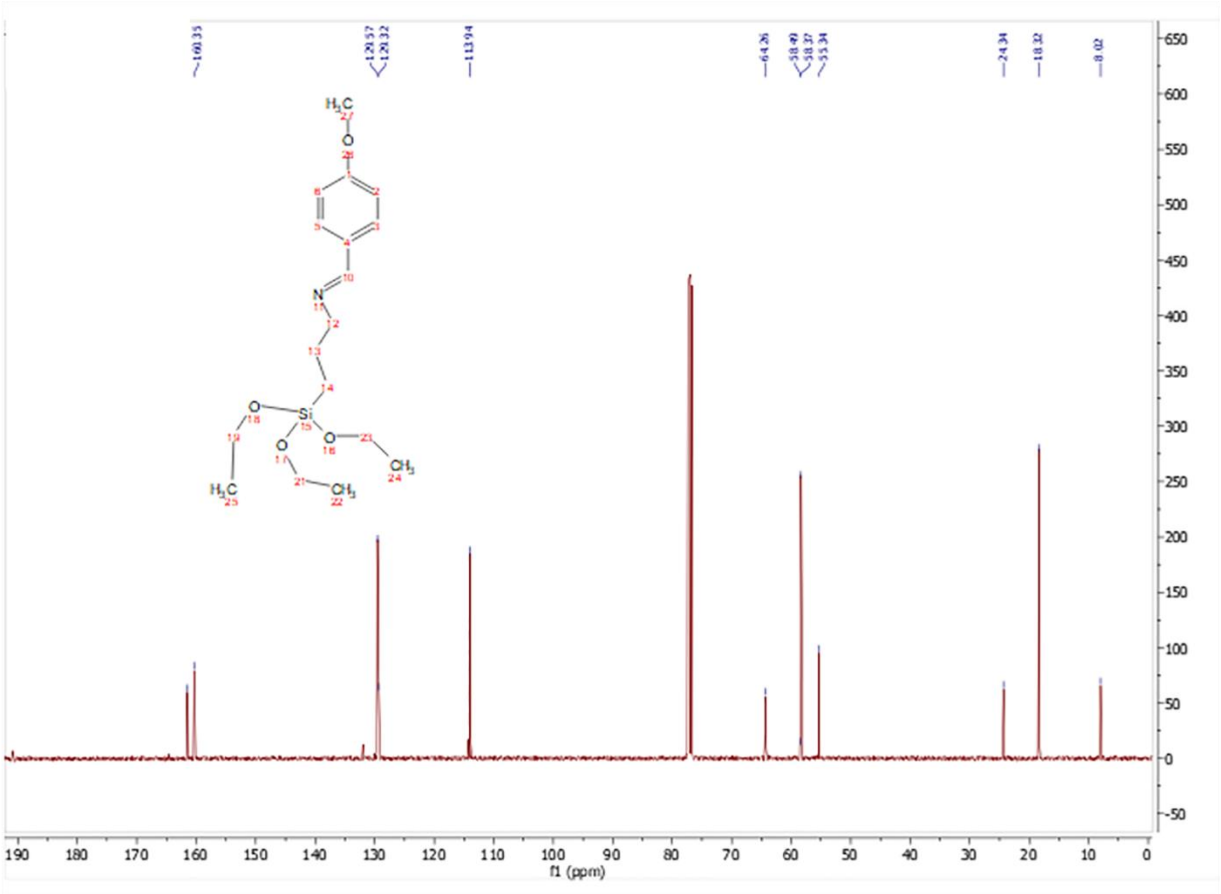

Fig. S21: ^{13}CNMR spectrum of MTPTM. ^{13}CNMR spectrum of (E)-1-(4-methoxyphenyl)-N-(3-(triethoxysilyl)propyl)methanimine.

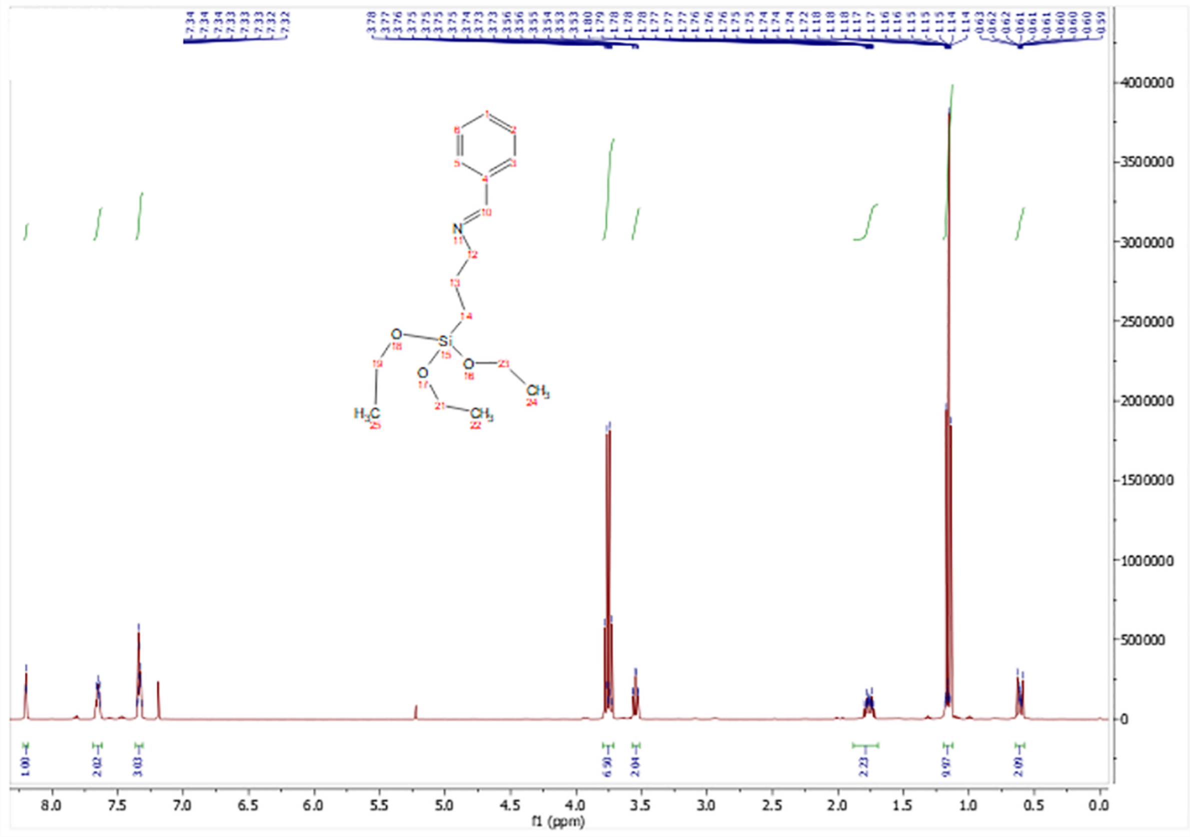

Fig. S22: ¹HNMR spectrum of PTM. ¹HNMR spectrum of (E)-1-phenyl-N-(3-(triethoxysilyl)propyl)methanimine.

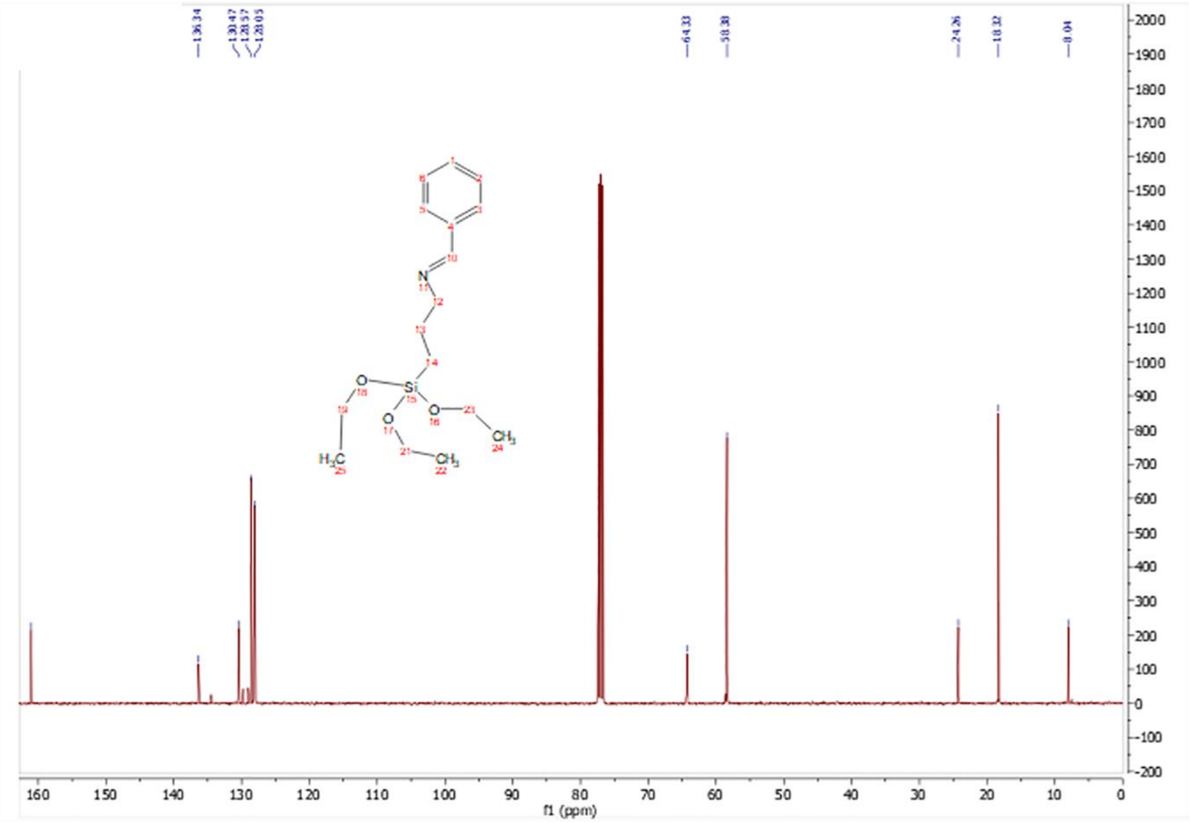

Fig. S23: ^{13}CNMR spectrum of PTM. ^{13}CNMR spectrum of (E)-1-phenyl-N-(3-(triethoxysilyl)propyl)methanimine.
